# Global Epigenetic Regulation of MicroRNAs in Multiple Myeloma

**DOI:** 10.1371/journal.pone.0110973

**Published:** 2014-10-17

**Authors:** Wenjing Zhang, Yaoyu E. Wang, Yu Zhang, Xavier Leleu, Michaela Reagan, Yong Zhang, Yuji Mishima, Siobhan Glavey, Salomon Manier, Antonio Sacco, Bo Jiang, Aldo M. Roccaro, Irene M. Ghobrial

**Affiliations:** 1 Department of Medical Oncology, Dana-Farber Cancer Institute, Harvard Medical School, Boston, Massachusetts, United States of America; 2 Nanfang Hospital, Southern Medical University, Guangzhou, China; 3 Center for Cancer Computational Biology, Dana-Farber Cancer Institute, Harvard Medical School, Boston, Massachusetts, United States of America; 4 The First People's Hospital of Yunnan Province, Kunming University of Science and Technology, Kunming, China; 5 Department of Hematology, Hopital Claude Huriez, Hospital of Lille (CHRU), Lille, France; Harbin Medical University, China

## Abstract

Epigenetic changes frequently occur during tumorigenesis and DNA hypermethylation may account for the inactivation of tumor suppressor genes in cancer cells. Studies in Multiple Myeloma (MM) have shown variable DNA methylation patterns with focal hypermethylation changes in clinically aggressive subtypes. We studied global methylation patterns in patients with relapsed/refractory MM and found that the majority of methylation peaks were located in the intronic and intragenic regions in MM samples. Therefore, we investigated the effect of methylation on miRNA regulation in MM. To date, the mechanism by which global miRNA suppression occurs in MM has not been fully described. In this study, we report hypermethylation of miRNAs in MM and perform confirmation in MM cell lines using bisulfite sequencing and methylation-specific PCR (MSP) in the presence or absence of the DNA demethylating agent 5-aza-2′-deoxycytidine. We further characterized the hypermethylation-dependent inhibition of miR-152, -10b-5p and -34c-3p which was shown to exert a putative tumor suppressive role in MM. These findings were corroborated by the demonstration that the same miRNAs were down-regulated in MM patients compared to healthy individuals, alongside enrichment of miR-152-, -10b-5p, and miR-34c-3p-predicted targets, as shown at the mRNA level in primary MM cells. Demethylation or gain of function studies of these specific miRNAs led to induction of apoptosis and inhibition of proliferation as well as down-regulation of putative oncogene targets of these miRNAs such as DNMT1, E2F3, BTRC and MYCBP. These findings provide the rationale for epigenetic therapeutic approaches in subgroups of MM.

## Introduction

Gene promoter DNA hypermethylation is one of the major epigenetic mechanisms responsible for silencing of tumor suppressor genes in a variety of malignancies [1], suggesting DNA methylation as a target for novel therapeutic agents. DNA methylation occurs at cytosine residues mainly in CpG islands, which represent specific genomic regions containing a high frequency of CpG sites [2]. Most CpG islands are located in the proximal promoter regions of genes and are usually methylated in tumor cells, mediating the inactivation of genes [3]. Recent studies have also highlighted the importance of miRNAs in supporting tumorigenesis [4]–[11].

MicroRNAs (miRNAs) are small non-coding RNAs of 19–25 nucleotides in length. In animals, miRNAs interact with specific target mRNAs, via complementary binding to sequences within the 3′ UTR, where they induce mRNA degradation or translational inhibition [5], [9]. In the vast majority of tumors, miRNAs are down-regulated in clonal cells, thus suggesting their ability to act as tumor suppressors [6], [7], [10], [11]. However, the mechanisms that control the expression of miRNAs are largely unknown. Prior studies have shown that miRNAs can be regulated by aberrant methylation of CpG islands encompassing miRNAs or adjacent to miRNAs [4]. For example, methylation-dependent silencing of the tumor suppressor-miR-127 and -124a has been identified in different tumor types [8], [12].

Aberrant promoter methylation has been described in multiple myeloma (MM) [13]–[17]. Specifically, p16 methylation represents one of the epigenetic aberrations that contribute to MM disease progression [18]. In addition, methylation-dependent silencing of multiple soluble Wnt antagonists, such as WIF1, DKK3, and APC has been reported in MM, thus explaining at least in part, the constitutive activation of Wnt signaling in clonal MM cells [19]. Moreover, epigenetic inactivation of the tumor suppressive miRmiR-194-2-192 cluster and miR-203 is implicated in the pathogenesis of MM [20], [21]. However, these studies highlight the methylation status of single genes or miRNAs, with highly variable prevalence of promoter hypermethylation within the same gene.

Herein, we used chromatin immunoprecipitation (ChIP) and array-based hybridization (ChIP-chip) to examine enrichment patterns of CpG islands in primary CD138^+^ bone marrow (BM) cells isolated from both MM patients and healthy donors (HD) and identified hypermethylation adjacent to miR-152, -10b-5p and miR-34c-3p. Moreover, by treating MM cells with a DNA demethylating agent 5-aza-2′-deoxycytidine (5-aza-CdR) we identified up-regulated miRNAs. Further validation experiments showed that miR-152, miR-10b-5p and miR-34c-3p were highly methylated, which may explain, at least in part, their low expression levels in MM. Finally, we showed that these three miRNAs act as putative tumor suppressor miRNAs by modulating several oncogenes in MM.

## Methods

### Cell lines and Tissue samples

The human MM cell lines MM.1S, RPMI 8226, OPM2, U266, IM9 and H929 were obtained from the American Type Culture Collection and grown in RPMI 1640 supplemented with 10% fetal bovine serum (FBS), 2 mM/ml L-glutamine, 100 U/mL penicillin, 100 µg/mL streptomycin (Invitrogen) as described previously [22]. Cells were treated with 5 µM of 5-aza-CdR (Sigma Aldrich) for 4 days, replacing the drug and medium every 24 hours [23]. Primary bone marrow stromal cells (BMSCs) were obtained from the bone marrow of patients with MM as described previously [22] and cultured in DMEM plus 20% FBS. Primary plasma cells were obtained from BM samples from patients with relapsed-refractory MM (N = 8). 5 males and 3 females were studied; median age 60 years old (range 48–75 years old). All the MM cases evaluated in these studies were patients with relapsed-refractory disease, who were off-therapy when bone marrow aspirates were collected. Previous therapies included either lenalidomide- or bortezomib-based regimens. Plasma cells were also collected from HD (N = 6). HD samples were then collected as 2 pooled control samples. Plasma cells were obtained using CD138^+^ microbead selection (Miltenyi Biotec, Auburn, CA) as previously described [22]. Approval for these studies was obtained from the Dana-Farber Cancer Institute Institutional Review Board. Informed consent was obtained from all patients and healthy volunteers in accordance with the Declaration of Helsinki protocol.

### ChIP-chip-based DNA methylation analysis

ChIP and ChIP-chip was performed according to described protocols [24] with some modifications. CD138^+^ plasma cells were obtained from MM patient bone marrow. 5 males and 3 females were studied; median age 60 years old (range 48–75 years old). All the MM cases evaluated in these studies were patients with relapsed-refractory disease, who were off-therapy when bone marrow aspirates were collected (i.e., at the time of progression). Previous therapies included either lenalidomide- or bortezomib-based regimens. CD138+ plasma cells were collected from 6 healthy donors and used for ChIP as 2 pooled controls. Cells were allowed to crosslink with 1% formaldehyde. After washing with ice-cold PBS, the cell suspension, in SDS lysis buffer, was disrupted by sonication on ice to gain genomic DNA fragments between 200 and 1000 bp. ChIP was performed with anti-5′-methyl-cytosine. Immunoprecipitated complexes were sequentially eluted and cross-link reversed. DNA fragments were purified using a PCR purification kit (Qiagen). The ChIPed DNA was amplified and hybridized to the Affymetrix GeneChip Human Tiling 2.0R Array Set as previously described [25]. DNA without immunoprecipitation was used as the input control. Microarray data was normalized by quantile normalization using Bioconductor [26]. The normalized data was then analyzed using the model-based analysis of tiling (MAT) array algorithm to identify genomic regions with the highest mean histone methylation scores [27]. Default parameters were used for each algorithm to find ChIP regions from all samples. The MAT library and mapping files were annotated with the nearest reference gene (1 kb distance) using the refseq annotation file based on Human Genome Assembly version 18 (hg18) which was downloaded from UCSC genome browser. Methylation peaks, with a fold change less than 10 compared to input, were filtered out for both MM and HD samples. Differential methylation was determined by the presence or absence of overlapping methylation peaks. Peaks that did not overlap with any peaks found in the contrasting condition were considered to be unique. The peaks were plotted using Circos (http://circos.ca/). MicroRNA coordinates were obtained from NanoString technologies (Seattle, WA), and the peaks overlapping microRNA regions were identified.

### miRNA isolation and microRNA expression analysis

miRNAs were isolated from six MM cell lines with or without 5-aza-CdR treatment (5 µM for 4 days) by miRNase mini kit (Qiagen) [28]. Quality control was done using RNA6000 Nano assay on the Aligent 2100 Bioanalyzer (Santa Clara, CA). miRNA detection was conducted using the nCounter human miRNA expression analysis system (Nanostring technologies, Seattle, WA) and performed according to the manufacturer's instructions. Briefly, 100 ng of miRNA was used as input material, with 3 µl of the threefold-diluted sample. A specific DNA tag was ligated onto the 3′ end of each mature miRNA, providing exclusive identification of each miRNA species in the sample. The tagging was performed in a multiplexed ligation reaction utilizing reverse complementary bridge oligonucleotides to dispose the ligation of each miRNA to its designated tag. All hybridization reactions were incubated at 64°C for 20 hours and then applied to the nCounter Preparation Station for automated removal of excel probes and immobilization of probe-transcript complexes on a streptavidin-coated cartridge. Data collection was carried out in the nCounter Digital Analyzer by counting individual fluorescent barcodes and quantifying target miRNA molecules present in each sample. Data normalization was performed by nSolver Analysis Software according to the manufacturer's instructions. Six internal positive spike controls were used to account for minor differences in hybridization and purification efficiencies, and six negative controls were used for the consideration of background hybridization.

### Bisulfate treatment and PCR conditions

Genomic DNA (gDNA) was isolated from MM cells with DNeasy Blood & Tissue Kit (Qiagen, Valencia, CA) as previously described [23]. 500 ng of gDNA was modified by treatment with sodium bisulfate using the EZ DNA Methylation-Gold Kit (Zymo Research, Orange County, CA), which induces chemical conversion of unmethylated cytosines into uracils, whit methylated cytosines being protected from this conversion [29]. The sequences of miRNA and its promoter were analyzed by using miRBase and the University of California at Santa Cruz Human Genome Browser (UCSC). CpG islands and specific primers for MSP and bisulfite-sequencing PCR (BSP) were designed by MethPrimer Tools (http://www.urogene.org/methprimer/) [30]. MSP analysis was performed with primers specific for either the methylated or unmethylated DNA. To verify sufficient DNA quality and successful DNA modification, human genomic DNA, with methylated CpG sites, was used as the positive control; H_2_O was used as the negative control. Amplified bisulfate PCR products were subcloned into the pGEM-T Easy vector (Promega, Madison, WI). Eight independent clones for each sample were selected and the T7 primers were used to sequence inserted fragments. Primers used are shown in File S1.

### Quantitative Real-time PCR

Quantitative Real-time PCR was performed as described previously [28]. Briefly, total RNA or miRNA from cells was isolated with RNeasy or miRNeasy Mini kit (QIAGEN). cDNA was synthesized using the SuperScript cDNA synthesis kit (Invitrogen) or High Capacity cDNA Reverse Transcription Kit (Life Technologies, Grand Island, NY) and quantitative RT-PCR (qRT-PCR) reactions were performed using SYBR MasterMix (Qiagen) by StepOnePlus Real-Time PCR System (Applied Biosystems, Foster city, CA) in triplicate. 18S or RNU6B was used to normalize the miRNA and mRNA data, respectively. Expression was calculated using the 2^−ΔΔCt^ method. Primers used are described in File S1.

### miRNA transfection

MM.1S, RPMI 8226, IM9 and H929 cell lines were transfected with hsa-miR-152, -10b-5p and -34c-3p mirVana miRNA or negative control mimics (Life Technologies, Grand Island, NY) at a final concentration of 50 nM, using Lipofectamine 2000 following manufacturer's instructions. Efficiency of transfection was validated by real-time PCR.

### Cell proliferation assays

Cell proliferation was measured by the incorporation of [^3^H] thymidine uptake assay (Perkin Elmer, Boston, MA), as described previously [22]. MM cells were incubated in 96-well plates transfected with miR-152, -10b-5p and miR-34c-3p and negative control mimics, respectively, following by being pulsed with [^3^H] thymidine (0.5 µCi/well) for at least 8 h of 48 h cultures. Cells were harvested onto glass filters with an automatic cell harvester (Cambridge Technology, Cambridge, MA), and counted using the LKB Betaplate scintillation counter (Wallac, Gaithersburg, MD). All experiments were performed in four-well repeat.

### Immunoblotting

Sodium dodecyl sulfate-polyacrylamidegel electrophoresis (SDS-PAGE) and western blotting were performed as previously described [23]. In brief, whole-cell lysates were separated using 10–12% gels and transferred to polyvinyldenefluoride (PVDF) membranes (Bio-Rad Laboratories, Hercules, CA). The antibodies used for immunoblotting included anti-PARP, -caspase 9, caspase 3 and α-tubulin (Cell Signaling Technology, Danvers, MA).

### Expression profiling and GSEA of available datasets

miR-152 and miR-10b-5p expression was analyzed using publicly available dataset where primary bone marrow selected CD138+ cells were obtained from either MM patients or HDs (GSE16558). Similarly, the expression levels of DNMT1, E2F3, BTRC and MYCBP were analyzed using publicly available datasets where primary bone marrow selected CD138+ cells obtained from either MM patients or healthy individuals were studied (GSE5900; GSE 2658) [31]. Gene Sets Enrichment Analysis (GSEA) was performed by using gene sets publicly available from the Broad Institute (Cambridge, MA; http://www.broadinstitute.org/gsea/index.jsp), as previously reported [32], [33]. Specifically, the following gene sets were used: TGCACTG, MIR-148A, MIR-152, MIR-148B; ACAGGGT, MIR-10A, MIR-10B and CACTGCC, MIR-34A, MIR-34C, MIR-449.

We used the easy-to-use graphical user interface of GSEA with gene set permutation to derive significance, with signal-to-noise as the distance metric and maximum expression to collapse probe sets to genes.

### Statistical analysis

miRNA expression data were normalized according to manufacturer's instructions (Nanostring technologies, Seattle, WA). To further define those miRNAs differentially expressed between groups (with vs without 5-aza-CdR treatment), the expression patterns of normalized data were analyzed and hierarchical clustering was performed using dChip (http://www.hsph.harvard.edu/cli/complab/dchip/) [34]. The enrichment analysis of targeted mRNAs of miR-152, -10b-5p and miR-34c-3p in GEO datasets was performed using GSEA, and considered significant with false discovery rate (FDR) <0.25, as previously reported (31). Mann-Whitney U rank ranksum test with GraphPad software was applied to describe the distribution of miRNAs and gene levels in MM patients compared with HDs. Statistical tests were unpaired, 2-sided t-tests comparing two conditions. *P* values less than 0.05 were considered significant. Data were presented for the purpose of figures as means and error bars.

## Results

### Global changes in DNA methylation occur in MM patients

**Figure 1:**
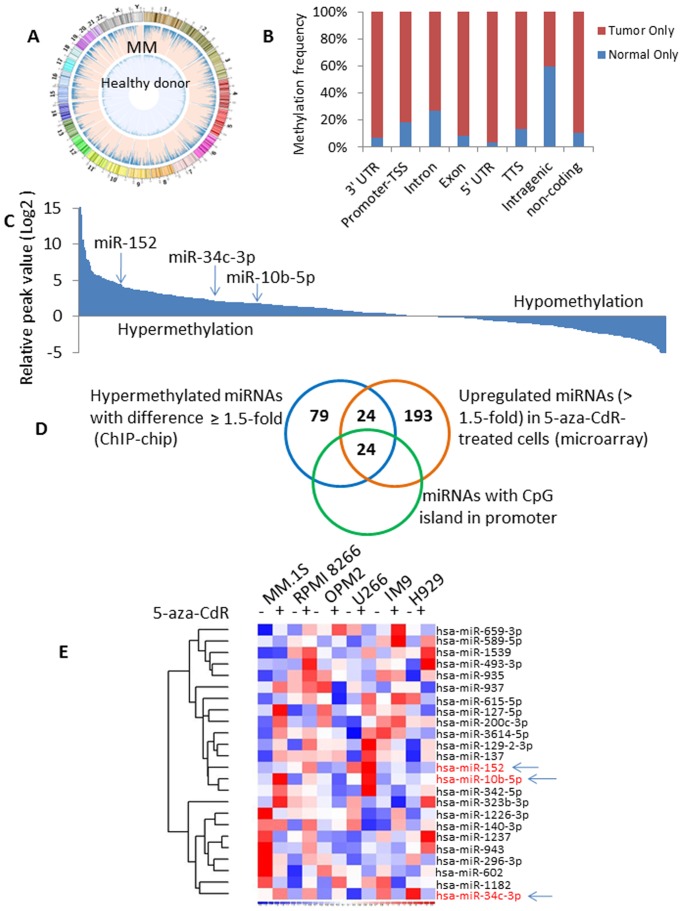
Methylation status of whole-genome sequencing data in MM and healthy donors. (A) Circular representation of DNA methylation levels in whole genome regions for MM and healthy donors (HD). (B) Genome-wide distribution of methylation peaks by gene regions in HD/MM only. (C) Methylation levels of regions around all known miRNAs in MM patients in decreasing order by peak intensity. Data was presented as log_2_ values. (D) Venn diagram compared ChIP-chip and miRNA microarray data, as well as miRNAs being present with CpG island. Left, miRNAs identified as high potential methylation with the definition of a difference ≥1.5-fold between MM and HD; Right: miRNAs up-regulated by more than 1.5-fold in at least two cell lines with 5-aza-CdR treatment; Bottom: miRNAs with CpG island. (E) Heatmap of the 24 overlay miRNAs in six MM cell lines with or without 5-aza-CdR treatment. Red, high expression; blue, low expression.

We first interrogated genome-wide methylation patterns in relapsed/refractory MM patients and HDs, and found a significant increase in global methylation in relapsed MM compared to HDs; circular representation of DNA methylation levels per each chromosome in MM and healthy donor is represented in Fig. 1A. The majority of methylation peaks were located in the intronic and intragenic regions in MM samples. Over half of the methylation peaks were identified within in intron regions (51.9% in HD and 59.6% in MM), without significant differences between HDs and MM. However, there were more significant regions differentially methylated in the 3′-UTR, promoter-TSS, exon, 5′-UTR, TTS and non-coding regions in MM compared with HDs (Fig. 1B). Of note, an 8.5-fold increase of non-coding regions was observed in MM compared to HDs. This leads us to hypothesize that methylation of miRNAs can be critical for the regulation of expression patterns of miRNAs in MM. We, therefore, mapped probe values of all known miRNAs to determine whether miRNA-related regions were differentially methylated in MM. As shown in Fig. 1C, there was a significant increase in the number of hypermethylated miRNAs compared to the number of miRNAs with hypomethylation. Specifically, 127 miRNAs were identified as highly methylated miRNAs with ≥1.5-fold change increase in MM compared to HDs (log_2_) (File S2). These findings suggested that miRNAs are highly methylated in MM and may therefore explain in part the global decrease in miRNA expression in these tumor cells.

To further examine the role of methylation in miRNA regulation in tumor cells, MM cell lines (MM.1S, RPMI 8266, OPM2, U266, IM9 and H929) were treated with the DNA methyltransferase inhibitor 5-aza-CdR and the global level of miRNAs was examined. Hierarchical clustering analysis of cell lines was performed and demonstrated up-regulation of miRNAs in response to treatment with 5-aza-CdR treatment with a>1.5-fold difference. Overall, 241 miRNAs were up-regulated in at least two cell lines with 5-aza-CdR (File S3), 77 miRNAs were up-regulated in three cell lines and 7 miRNAs were up-regulated in four cell lines treated.

To further identify the most critically regulated miRNAs, we identified 48 miRNAs that were present in the methylation sites of MM patient samples and were also up-regulated in response to 5-aza-CdR treatment in MM cell lines (Fig. 1D). Among them, 4 miRNAs, miR-3605-5p, -4461, -4516 and miR-4531, were not present in UCSC and were excluded; 44 miRNAs remained. We then assessed the status of CpG islands for these 44 miRNAs and found that only 24 miRNAs were present in one or more CpG island upstream regulatory sequences (Fig. 1D&E).

### Identification and expression analysis of miRNA candidates in MM

Among the 24 miRNAs, miR-152 was the most frequently up-regulated miRNA with 5-aza-CdR treatment (in all six cell lines). In addition, miR-10b-5p was up-regulated by more than 2.0-fold in at least three cell lines following treatment with 5′-aza-CdR. Therefore, we further examined the functional significance of miR-152 and miR-10b-5p in parallel with miR-34c-3p, which was also included in the 24 miRNAs and has been reported to be methylated in MM [35].

**Figure 2:**
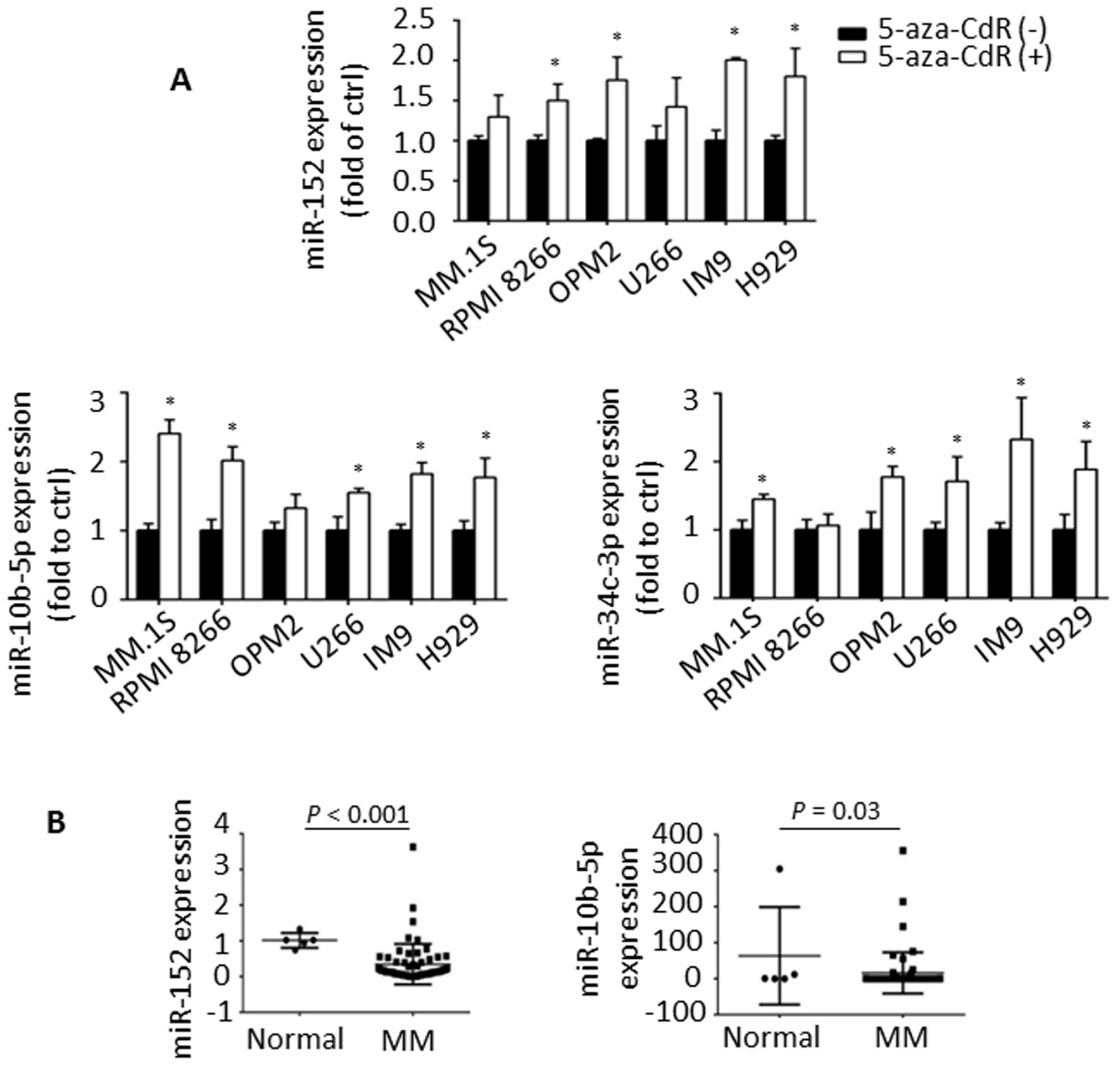
Identification and expression of miRNA candidates in MM. (A) Effect of 5-aza-CdR on the expression of miR-152, -10b-5p and miR-34c-3p in six MM cell lines by real-time PCR. Experiments were performed in triplicate and RNU6B was used as the internal control. Data were shown as means ± SD. ^*^ *P*<0.05 compared with cells without 5-aza-CdR. (B) Expression levels of miR-152 and miR-10b-5p in MM and healthy donor in GSE16558.

Further validation of the re-expression of these miRNAs was performed by real-time PCR and indeed confirmed that miR-152, -10b-5p and miR-34c-3p were significantly increased following exposure to 5-aza-CdR (Fig. 2A). Of note, all of the regions around these three miRNAs were highly methylated in MM patients as previously shown in Fig. 1C. We next analyzed the expression levels of these miRNAs in MM using GSE16558, and found that both miR-152 and miR-10b-5p were significantly down-regulated in MM patients compared to HD (Fig. 2B).

### Methylation analyses of miR-152, -10b-5p and miR-34c-3p in MM cells

**Figure 3:**
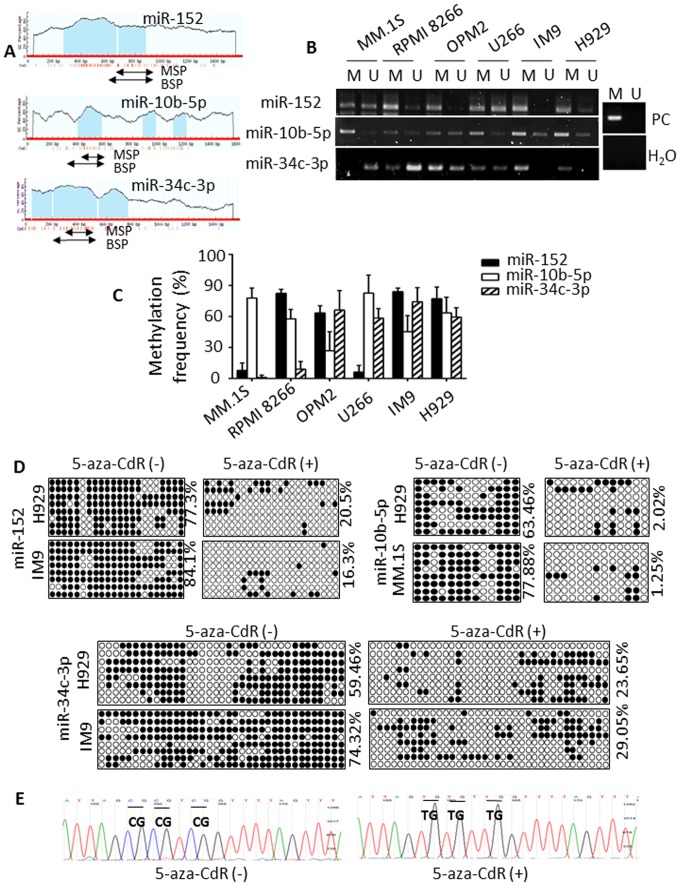
Methylation of miR-152, -10b-5p and miR-34c-3p in MM cells. (A) Schematic illustration of the percentage of C + G nucleotides (CG%) and the density of CpG olinucleotides were shown for a region spanning 1000 bp upstream and 500 bp downstream of miR-152, miR-10b-5p and miR-34c-3p, respectively. Specific primers for these CpG islands were designed (arrows) and used to amplify these DNA fragments in MM cell lines. The CpG island was depicted, and each vertical bar illustrated a single CpG. (B) Representative MSP results of the three miRNAs methylation inMM1S, RPMI 8266, OPM2, U266, IM9 and H929 cell lines. M: methylated primers; U: unmehtylated primers. PC: positive control. (C) Bisulfite sequencing analysis showed relative methylation frequencies of miR-152, -10b-5p and miR-34c-3p in six MM cell lines. Eight single clones for each sample were selected and T7 primers were used for sequencing. (D) Bisulfite sequencing analysis showed methylation frequencies of miR-152 and miR-34c-3p in H929 and IM9, and miR-10b-5p in H929 and MM1S, treated with or without 5′-aza-CdR (5 µM) for 4 days. Black and white circle represented methylated and unmethylated CpG, respectively. (E) Representative sequencing results showed that the cytosine (C) residues of CpG dinucleotides were converted into thymidine (T).

miR-152, -10b-5p and miR-34c-3p present with 2, 3 and 3 distinct CpG islands in their upstream chromosomal regions, respectively (1000 bp upstream and 500 bp upstream) as shown in Fig. 3A. We further examined the methylation status of these miRNAs using MSP analysis and showed that the miR-152 upstream promoter region was methylated in RPMI 8266, OPM2, IM9 and H929 cells; while partial/no methylation was detected in MM.1S and U266 cells. Moreover, miR-10b-5p methylation was observed in all MM cell lines tested. Complete methylation of miR-34c-3p was observed in IM9 and H929; while partial methylation was found in OPM2 and U266; in contrast with MM.1S and RPMI 8266 that were unmethylated (Fig. 3B). To demonstrate the frequency of CpG island methylation in these three miRNAs, we undertook bisulfite sequencing of multiple clones (Fig. 3C). Detailed analysis has been provided in Table 1. These results support that miR-152, miR-10b-5p and miR-34c-3p hypermethylation frequently occurs in MM cells. In addition, the results of MSP and BSP suggest that the methylation of miRNAs is cell-type specific.

**Table 1:** Methylation frequency of miR-152, -10b-5p and miR-34c-3p in MM cell lines.

	Methylation frequency (%)					
	MM1S	RPMI 8266	OPM2	U266	IM9	H929
miR-152	8.0	82.4	63.6	6.3	84.1	77.3
miR-10b-5p	77.9	57.7	26.9	82.7	45.2	63.5
miR-34c-3p	1.2	9.2	66.2	58.5	74.3	59.5

To determine whether 5-aza-CdR may modulate miRNA methylation status in MM cells, bisulfite sequencing was further performed in H929, IM9 and MM1S cells for specific miRNAs respectively, which was based on their different methylation frequencies in each cell line. As shown in Fig. 3D, the methylation frequencies of miR-152 decreased from 77.3% to 20.5% in H929, and 84.1% to 16.3% in IM9 cells after 5-aza-CdR treatment. Similar results were found for miR-10b-5p in H929 (63.46% to 2.02%) and MM1S (77.88% to 1.25%) cells, as well as for miR-34c-3p in H929 (59.46% to 23.65%) and IM9 (74.32% to 29.05%). The representative sites with the conversion from cytosines (C) to uracil (T) are shown in Fig. 3E. Taken together, these findings suggest that the CpG-rich promoter regions of miR-152, -10b-5p and miR-34c-3p are hypermethylated and present with low expression in MM cells.

### MiR-152, -10b-5p and miR-34c-3p act as tumor suppressors in MM

**Figure 4:**
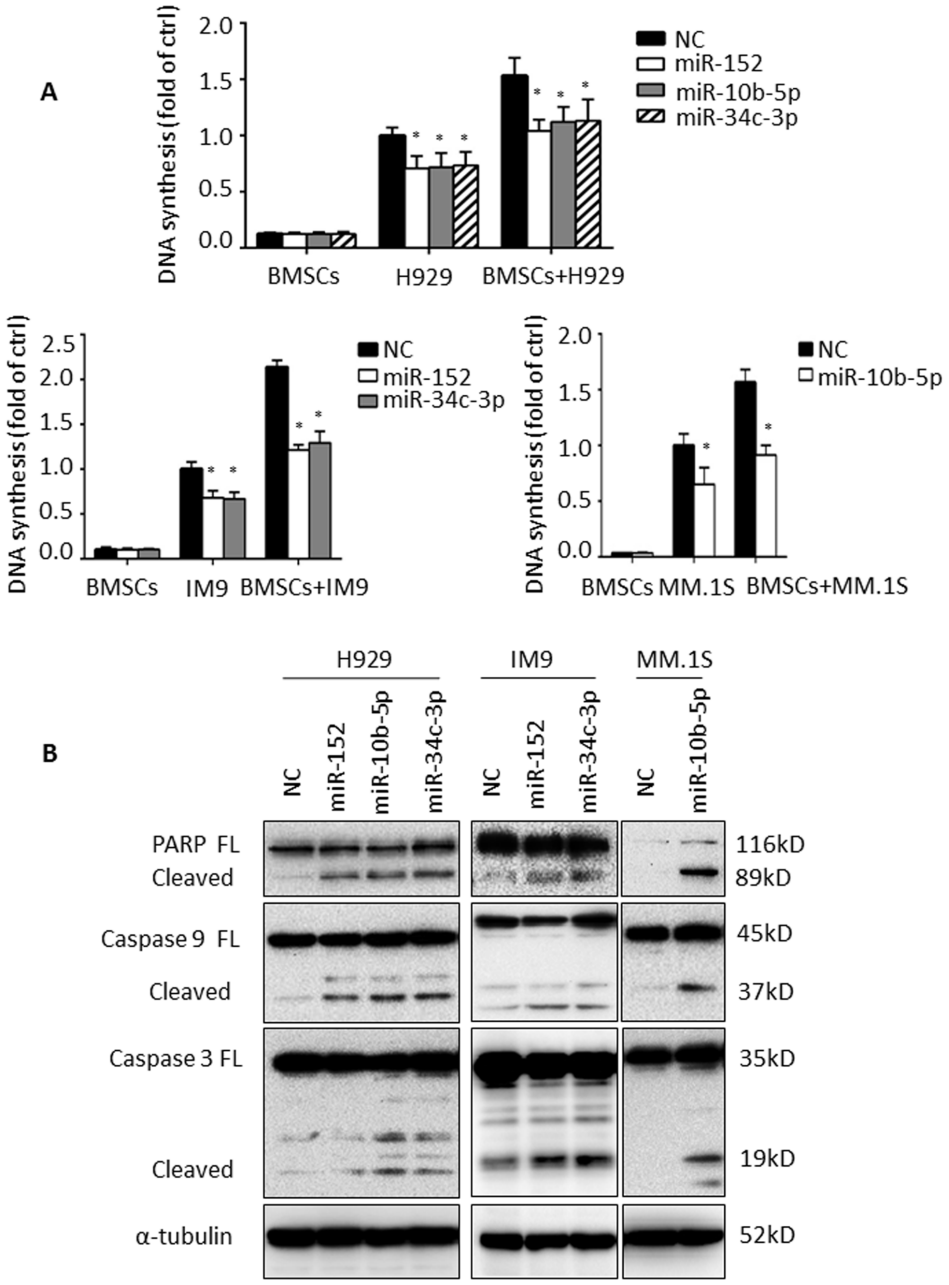
miR-152, -10b-5p and miR-34c-3p modulated proliferation and apoptosis of MM cells. Since the three miRNAs have different methylation frequencies and expression levels in MM cell lines, H929 cells were transfected with miR-152, -10b-5p and -34c-3p mirVana miRNA respectively; IM9 cells were transfected with miR-152 and -34c-3p mirVana miRNA respectively; MM1S cells were used to overexpress miR-10b-5p. All of the cells transfected with negative control mimics used as control cells. (A) The indicated transfected cells were cultured in presence or absence with primary BMSCs for 72 hours, following by [H]^3^ thymidine uptake assay. NC-transfected cells in absence with BMSCs were defined as 1.0 and regarded as the control group. ^*^ *P*<0.05. (B) MM cells (NC-, miR-152-, -10b-5p-, or miR-34c-3p-transfected) were harvested at 48 or 72 hours after transfection. Whole cell lysates were subjected to western blotting using anti-PARP, -Caspase 9, -Caspase 3 and -α-tubulin antibodies.

We next examined the potential functional relevance of miR-152, -10b-5p and -34c-3p in MM. We therefore, examined the role of these 3 miRNAs in the growth of tumor cells alone or in the presence of bone marrow stromal cells (BMSCs) using gain of function studies. Efficiency of transfection with miR-152, -10b-5p and miR-34c-3p was first demonstrated using real-time PCR (Figure S1). There was a significant inhibition of proliferation of MM cells in response to re-expression of miR-152, -10b-5p and -34c-3p in MM cells, even in the presence of BMSCs, indicating that these miRNAs are critical for the growth and proliferation of MM cells even in the presence of the bone marrow microenvironment (Figure 4A, *P*<0.05). The effects of these miRNAs in modulating MM cell apoptosis was also investigated; re-expression of miR-152, -10b-5p and miR-34c-3p mimics induced cleavage of PARP- and caspase-9 and -3, compared to normal control-transfected cells (Fig. 4B). These findings suggest that miR-152, -10b-5p and miR-34c-3p may act as putative tumor suppressors in MM by inhibiting cell proliferation and inducing apoptosis.

### MiR-152 and miR-10b-5p mediate the activation of oncogenic target genes

**Figure 5:**
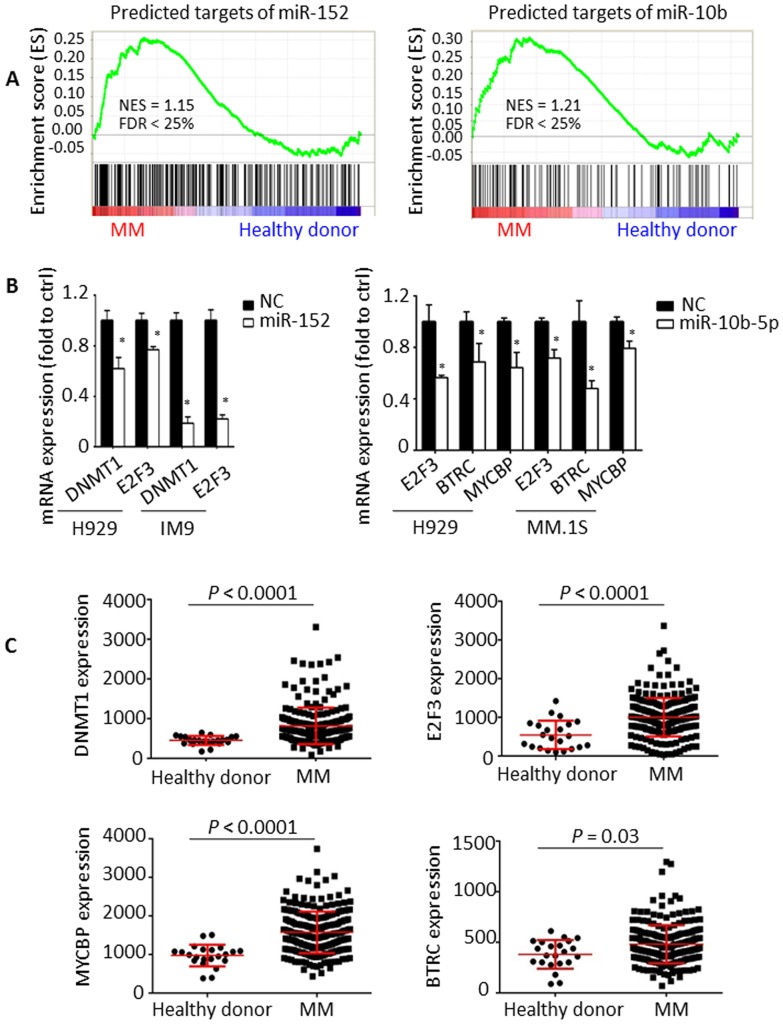
Effect of miR-152 and miR-10b-5p on the expression of putative targets. (A) GSEA established that predicated targets of miR-152 and miR-10b-5p was positively correlated with MM, and negatively correlated with HD. NES: normalized enrichment score; FDR: false discovery rate. (B) The expression of predicted targets of miR-152 (DNMT1 and E2F3) and miR-10b-5p (E2F3, BTRC and MYCBP) in miR-152-, -10b-5p- or NC-transfected MM cells by real-time PCR with normalization to the reference 18S expression. ^*^ *P*<0.05 compared with NC-transfected cells. (C) The expression of DNMT1, E2F3, BTRC and MYCBP in healthy donor and MM (GSE5900 and GSE2658).

Given that miRNAs inhibit mRNA expression, we hypothesized that the predicted target genes of these specific miRNAs are highly expressed in CD138^+^ bone marrow-derived MM cells. We therefore screened publically available mRNA datasets (GSE5900; GSE2658) and found that MM patients present with a significant enrichment of miR-152- and miR-10b-target genes, as shown by GSEA (FDR<0.25; Fig. 5A), whereas no significant differences were observed for miR-34c-predicted targeted genes. We next focused on the predicted genes of miR-152- and miR-10b-5p. DNA-methyltransferase 1 (DNMT1), a predicted target of miR-152; beta-transducin repeat containing E3 ubiquitin protein ligase (BTRC) and Myc binding protein (MYCBP), predicted targets of miR-10b-5p and E2F transcription factor 3 (E2F3), predicted target of both miR-152 and miR-10b-5p were examined in MM cells (Figure S2). Enhanced expression of miR-152 induced a significant reduction of DNMT1 and E2F3 expression. In addition, E2F3, BTRC and MYCBP were significantly increased after miR-10b-5p mimic transfection (Fig. 5B). Moreover, we confirmed that the expression of these genes is significantly up-regulated in MM patients compared to healthy donors using GSE5900 and GSE2658 as shown in Fig. 5C. These results suggest that methylation of miR-152 and miR-10b-5p enhances MM progression through increased expression of specific oncogenes, such as DNMT1, BTRC, MYCBP and E2F3.

## Discussion

Aberrant promoter hypermethylation has been described in tumors for specific gene clusters in MM [4], [35]. In addition, recent studies have shown that MM is characterized by highly variable DNA methylation patterns that exceed the methylation variability seen in several solid cancers, with focal hypermethylation changes in clinically aggressive subtypes, such as plasma cell leukemia and patients with translocation t(4;14), suggesting that methylation changes can affect disease biology [36]. Moreover, Kaiser et al [16] identified epigenetically repressed tumor suppressor genes that were associated with prognostic relevance in myeloma. In our study, we found that the majority of methylation peaks were located in the intronic and intragenic regions in MM samples. Therefore, we investigated the effect of methylation on miRNA regulation in MM.

miRNAs act as tumor suppressors or oncogenic activators in many cancers, however, the majority of cancers present with global suppression of miRNAs including MM [6], [7], [10], [11], [36]–[38]. To date, the mechanism of global miRNA suppression in cancers, and specifically in MM, has not been investigated. In this study, we showed that hypermethylation occurred in regions of miRNAs in MM, compared to HDs indicating that miRNAs are highly methylated in MM, which could explain the global decrease of miRNA expression in MM cells. These studies were confirmed in MM cell lines using bisulfite sequencing and MSP in the presence or absence of the demethylating agent 5-aza-CdR.

Our studies describe the presence of hypermethylation-dependent inhibition of miR-152, -10b-5p and -34c-3p, which were shown to exert a putative tumor suppressive role in MM. We identified these miRNAs though global methylation studies in patient samples as well as providing functional validation in MM cell lines with bisulfite sequencing and MSP in the presence or absence of 5-aza-CdR. These findings were corroborated by the demonstration that the same miRNAs were present at lower level in MM patients compared to healthy individuals, together with enrichment of miR-152-, -10b-5p, and -34c-3p-predicted targets as shown at the mRNA level in primary MM cells. Demethylation or re-expression of these specific miRNAs led to induction of apoptosis and inhibition of proliferation as well as down-regulation of putative oncogene targets of these miRNAs.

MiR-10b may exert a bifunctional role, as shown by its activity as oncogene or tumor suppressor depending on the specific tumor type. For instance, it may positively regulate cell invasion and metastasis in breast cancer through Twist modulation [39], [40]. In pancreatic cancer, miR-10b has been reported to enhance cell invasion by suppressing TIP30 expression and promoting EGF- and TGF-β-mediated pathways and it can be also considered as a novel diagnostic biomarker [41]. In contrast, miR-10b has been shown to play a tumor suppressive role in gastric cancer and endometrial serous adenocarcinomas [42], [43]. In our studies, we found that miR-10b-5p (previous ID: miR-10b) was down-regulated via promoter methylation, resulting in inhibition of MM cell proliferation. Importantly, methylation of miR-10b-5p occurred in the MM patient samples based on a global methylation analysis [16]. These findings suggest that miR-10b-5p is regulated through different mechanisms, such as transcription regulation and promoter methylation, thereby presenting different expression profiles and functions in different types of tumors.

Aberrant methylation of miR-152 has been reported in both solid tumors and hematological malignancies. Previous studies show that miR-152 is methylated and inhibits cell growth and proliferation in breast and endometrial cancer [44], [45]. In addition, methylation of miR-152 contributes to its down-regulation in hepatitis B virus-related hepatocellular carcinoma (HCC) [46]. miR-152, among others, is down-regulated in t(4;11) positive ALL as a consequence of CpG methylation [47]. Moreover, an association between miR-152 expression and lower survival in patients with MM has been previously demonstrated [48]. Interestingly, it has been reported miR-152 is down-regulated in hyperdiploid MM compared with non-hyperdiploid disease, leading to the up-regulation of several oncogenes [49].

Since each single miRNA has a large number of predicted or established target genes, we selected several putative oncogenes to study, such as DNMT1, E2F3, BTRC and MYCBP. Among them, DNMT1 is a major enzyme responsible for maintenance of DNA methylation patternspatter. Its aberrant expression is the dominant mechanism for the genome instability, which associates with tumorigenesis and cancer development [50]. E2F3 functions as a transcription factor which is involved in the regulation of cell proliferation [51]. In fact, it has been observed that DNMT1 and E2F3 are candidate targets of miR-152 in HCC, endometrial and breast cancer [44]–[46]; BTRC has been shown to ubiquitinate phosphorylated NFKBIA, targeting it for degradation and thus activating NF-κB [52]. MYCBP is able to enhance c-Myc'sMyc ability to activate E box-dependent transcription [53]. The activation of both NF-κB and c-Myc partly contributes to the progression of MM. In our study, we found that down-regulation of miR-152 and miR-10b-5p is supported by over-expression of DNMT1, E2F3, BTRC and MYCBP in MM patients. More importantly, ectopic expression of miR-152 or miR-10b-5p significantly down-regulated the mRNA level of these genes. Hence, we conclude that MM cell proliferation may be mediated, at least in part, through methylation and down-regulation of miR-152- and miR-10b-5p which in turn lead to activation of DNMT1, E2F3, BTRC and MYCBP.

In conclusion, our studies indicate that global miRNA suppression in MM may be due to hypermethylation of non-coding regions in the MM genome. More specifically, miR-152, -10b-5p and miR-34c-3p are epigenetically silenced in MM through CpG island methylation; and act as potential tumor suppressor miRNAs in this disease. Re-expression of these miRNAs led to suppression of oncogenes and the inhibition of proliferation and induction of apoptosis in MM cells. These findings establish an important mechanism of miRNA deregulation in MM. Specifically, we suggest that miR-152, -10b-5p and miR-34c-3p promoter methylation may represent useful molecular biomarkers for assessing the risk of MM development. Most importantly, our study might provide a mechanistic and molecular basis for a new therapeutic use for pharmacological compounds with DNA demethylating activity in the treatment of MM patients.

## Supporting Information

Figure S1 **Validation of miRNA expression by real-time PCR.** The indicted cells were transfected with miR-152, -10b-5p, -34c-3p or negative control (NC) respectively. miRNA levels were detected by real-time PCR, and normalized to RNU6B control. Data were shown as means ± SD. ^*^ *P*<0.05 compared with NC-transfected cells.
(TIF)

Figure S2 **Binding of miR-152 or miR-10b-5p to the indicated target mRNAs at different sites.** Complementary sites between miR-152 and DNMT1 and E2F3, and between miR-10b-5p and E2F3, BTRC and MYCBP according to Targetscan 6.2.
(TIF)

File S1 **List of primer sequences and PCR products.**
(DOC)

File S2 **MM patients present with higher miRNA methylation as compared to healthy individuals.**
(XLSX)

File S3 **5-aza-CdR-dependent modulation of miRNAs in MM cell lines.**
(XLS)
