# Supplementary figures and images for: Global Epigenetic Regulation of MicroRNAs in Multiple Myeloma
Source: PLoS One. 2014 Oct 17;9(10):e110973. doi: 10.1371/journal.pone.0110973 (PMC4201574; doi:10.1371/journal.pone.0110973)

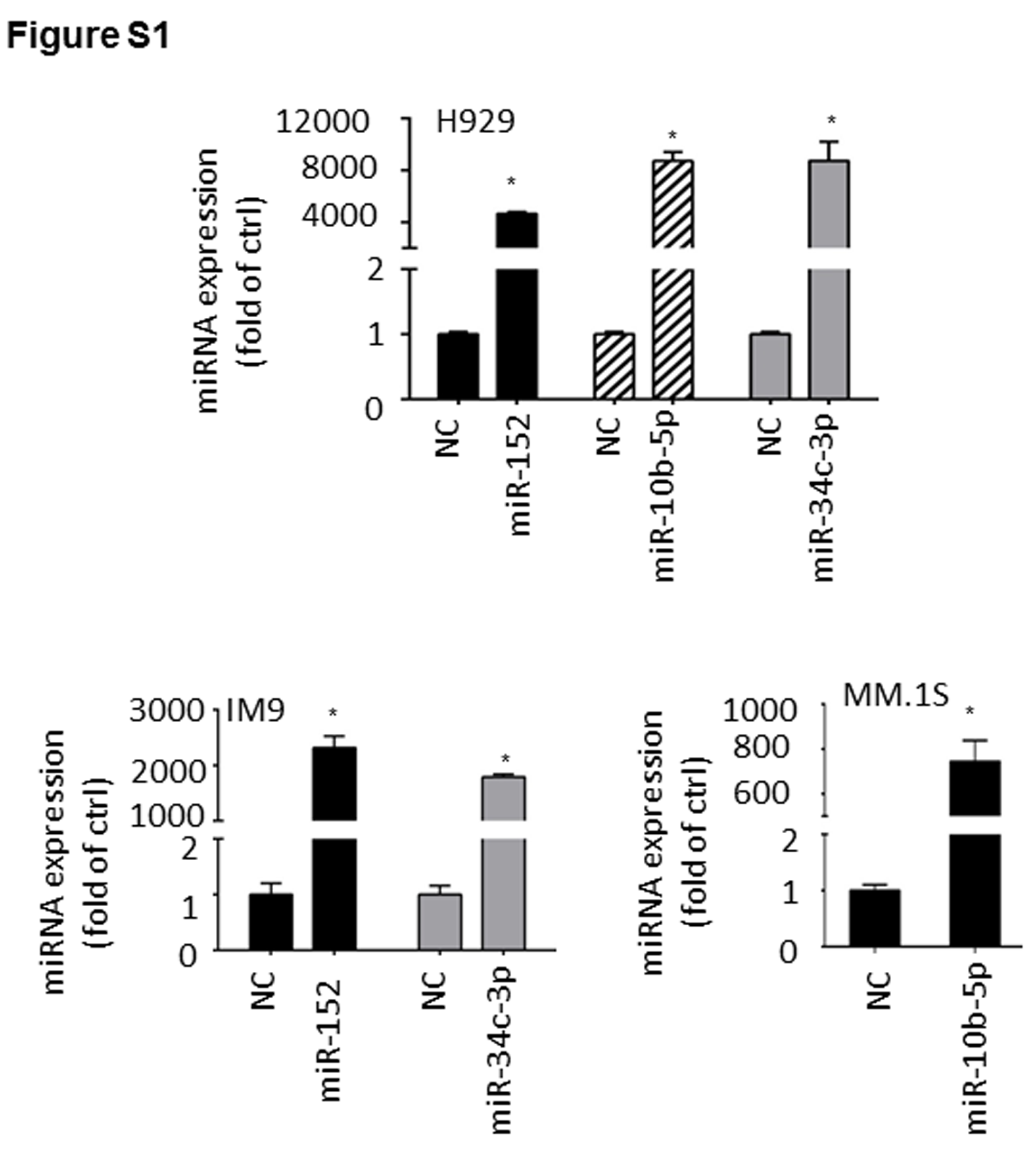

Supplement: Figure S1 — Validation of miRNA expression by real-time PCR. The indicted cells were transfected with miR-152, -10b-5p, -34c-3p or negative control (NC) respectively. miRNA levels were detected by real-time PCR, and normalized to RNU6B control. Data were shown as means ± SD. * P<0.05 compared with NC-transfected cells. (TIF) [file pone.0110973.s001.tif]
