# Supplementary material for: Global Epigenetic Regulation of MicroRNAs in Multiple Myeloma
Source: PLoS One. 2014 Oct 17;9(10):e110973. doi: 10.1371/journal.pone.0110973 (PMC4201574; doi:10.1371/journal.pone.0110973)
Supplement: File S1 — List of primer sequences and PCR products. (DOC) [file pone.0110973.s003.doc]

**FILE S1. Primer sequences and PCR products.**

|  | miRNA | Primer sequence | PCR product (bp) |
| --- | --- | --- | --- |
| MSP | miR-152-M | TTATTTTTGATTGGTTTTAGGATTC | 273 |
|  |  | CTAACCGAACTAAACCTACGCT |  |
|  | miR-152-U | TATTTTTGATTGGTTTTAGGATTTG | 275 |
|  |  | TCCCTAACCAAACTAAACCTACACT |  |
|  | miR-10b-5p-M | TCGTTTTGTTTTTTATTATTAGCGC | 111 |
|  |  | GAACTAACCTCTCCGTTCCG |  |
|  | miR-10b-5p-U | ATTTTTTGTTTTGTTTTTTATTATTAGTGT | 117 |
|  |  | CAAACTAACCTCTCCATTCCACT |  |
|  | miR-34c-3p-M | GTTCGTTGGTTTAGTTACGC | 179 |
|  |  | GACTACAACTCCCGAACGAT |  |
|  | miR-34c-3p-U | GTTTGTTGGTTTAGTTATGTGT | 180 |
|  |  | CAACTACAACTCCCAAACAAT |  |
| BSP | miR-152 | AGAGTAGGGTTAGGGGGAGTAGTTA | 286 |
|  |  | ATAAACTCCAAAAACATACCCATCA |  |
|  | miR-10b-5p | TTTTTGTATTTTTGTGTGTTGTTGT | 220 |
|  |  | CATTTACCTTCATAAACCCTAATCC |  |
|  | miR-34c-3p | GTTTGGTATTTTTGGGGGTTAT | 312 |
|  |  | ACCACAATACAATCAACTAATAACACTAC |  |
| Real-time PCR | DNMT1 | CGGCCTCATCGAGAAGAATATC | 135 |
|  |  | AAGCCAGTGATCCACCATTC |  |
|  | E2F3 | GGGCCCATTGAGGTTTACTTAT | 109 |
|  |  | AGTCTTTGGAAGCGGGTTTAG |  |
|  | TFAP2C | CCAGTCTTGGAGACGAACATAC | 130 |
|  |  | TGACAATCAGGGCTTCTTTGA |  |
|  | ZMYND11 | GAGCAGCTAAAGGTCACTCAA | 135 |
|  |  | CTGAGGCATCGTGGGTATTT |  |
|  | BTRC | TGGCTCATCTGACAACACTATC | 96 |
|  |  | CGAATACAACGCACCAATTCC |  |
|  | MYCBP | CCAAGGTGTTGGTAGCCTTAT | 120 |
|  |  | CTAGGCGAAGCAGCTCTATTT |  |
